# Stability of Cybergrooming Victimization Among Adolescents: A One-Year Latent Transition Analysis

**DOI:** 10.1007/s10964-026-02333-w

**Published:** 2026-03-19

**Authors:** Catherine Schittenhelm, Manuel Gámez-Guadix, Sebastian Wachs

**Affiliations:** 1 Institute of Education, University of Münster, Münster, Germany; 2 Department of Biological and Health Psychology, Autonomous University of Madrid, Madrid, Spain

**Keywords:** Cybergrooming, Online grooming, Sexual victimization, Victimization stability, Latent transition analysis

## Abstract

Given the high prevalence and potential harms of cybergrooming, understanding distinct patterns and the stability of victimization experiences among adolescents is imperative. Therefore, this one-year longitudinal study examined latent classes of cybergrooming and their stability. The sample consisted of 688 Spanish adolescents (56% girls, 88.5% heterosexual) with a mean age of 13.86 years (*SD* = 1.22). At both time points, latent class analysis with experienced grooming strategies as binary indicators supported a three-class solution: no-victimization, low-victimization, and high-victimization. The victimized classes were highly similar in their pattern of experienced strategies, except that, in contrast to the low-victimization class, sexualization was the most likely strategy to experience in the high-victimization class. Further, based on most likely class membership, experiencing multiple strategies was more prevalent in the high-victimization class than in the low-victimization class. Subsequent analyses accounting for classification uncertainty indicated that the three classes differed significantly on (1) selected socio-demographic variables, namely gender, age, and sexual orientation, and (2) psychosocial variables, namely depressive symptoms and emotional involvement, but not social support. Latent transition analysis revealed that, for example, higher age and higher depressive symptoms increased the risk of transitioning from the no- to the high-victimization class. Stability was particularly high in the no- and high-victimization classes. Limitations of the present study include the dichotomization of grooming items for the main analyses and a slight inconsistency in the classification of certain patterns of experienced strategies across time. Overall, these findings indicate that a subset of adolescents is particularly vulnerable to experiencing multiple strategies and chronic or recurring victimization.

**Supplementary Information:**

The online version contains supplementary material available at 10.1007/s10964-026-02333-w.

## Introduction

Information and communication technologies (ICTs) are omnipresent in the daily lives of young people, which exposes them to several online risks, including cybergrooming victimization (Machimbarrena et al., 2018). In this type of sexual online victimization, perpetrators employ various grooming strategies to manipulate young people to establish a sexually exploitative relationship (Wachs et al., 2012). As cybergrooming victimization can significantly impair victims’ psychological well-being (e.g., Ortega-Barón et al., 2022; Whittle et al., 2013), it is not only crucial to prevent young people from being victimized in the first place (i.e., identifying risk factors informing prevention programs) but also to gain insights into which factors contribute to the continuity of victimization experiences or the risk of revictimization. While first longitudinal studies have shown that for some cybergrooming victims, the victimization experience can in fact be stable or recurring over a prolonged period of time (Gámez-Guadix et al., 2023; Ortega-Barón et al., 2022), the current state of research lacks information on factors related to the stability of victimization. A second research gap concerns whether there are distinct subgroups of cybergrooming victims. Building on research on cybergrooming perpetrators, which implies perpetrator typologies depending on, e.g., their employed grooming strategies (Moosburner et al., 2025), there may also be subgroups of victims who experience distinct patterns of grooming strategies. Therefore, the present study focused on (1) identifying potential subgroups of victims based on the grooming strategies they encountered, and (2) analyzing the stability of victimization and factors influencing it, partially guided by an adapted version of the General Aggression Model (Anderson and Bushman, 2002; Schittenhelm et al. 2025a). This provides, for example, insights into whether some victim groups are particularly vulnerable to stable victimization trajectories, and information for prevention and intervention efforts on which factors need to be considered when aiming to destabilize victimization experiences.

### Definition, Prevalence, and Stability of Cybergrooming Victimization

Cybergrooming refers to a form of sexual victimization of children and adolescents that involves perpetrators using ICTs to solicit and exploit minors for sexual purposes (Wachs et al., 2012). This phenomenon differs conceptually from other forms of online sexual victimization, such as sexual solicitation, as more singular, unidirectional attempts at sexual contact (Gandolfi et al., 2021). Rather, cybergrooming denotes a psychologically manipulative process in which perpetrators systematically establish a relationship with their victims. To this end, perpetrators employ various grooming strategies (e.g., Moosburner et al., 2025; Ringenberg et al., 2022). Five common strategies are deception, gift-giving, interest in the victim’s environment, sexualization, and aggression (Gámez-Guadix et al., 2021). *Deception* involves feigning common interests and, at least initially, giving false information about identity characteristics such as age or physical appearance (Quayle et al., 2014). *Gift-giving* refers to both offers with and without explicit requests for consideration. Perpetrators may use this strategy as a door opener for sexual interactions by requesting, for example, sexually explicit photos or videos in exchange for money or other currencies (e.g., items in the online gaming context). *Interest in the victim’s environment* refers to a strong focus on the victim’s social network, everyday life, and well-being. This strategy is central to establishing a relationship with the victim but may also help assess and minimize the risk of detection (Moosburner et al., 2025). *Sexualization* of the conversation refers to the introduction of sexual content, for instance, through sexual comments, compliments, or questions. While this sexualization may occur gradually, some perpetrators employ sexualizing strategies very soon after the conversation begins (Kloess et al., 2019). Finally, *aggression*, including harassment, intimidation, and coercion, may be employed by perpetrators to maintain the relationship and the victim’s compliance or to escalate sexual behaviors (Moosburner et al., 2025).

Accumulated research indicates that cybergrooming is a prevalent phenomenon among adolescents. Drawing from self-report data, a systematic review showed that most reported prevalence rates among adolescents ranged from 10% to 20% (Schittenhelm et al., 2025a). These prevalence rates mostly referred to reference periods of up to 1 year, whereas studies examining longer periods reported rates of 30–40% (Gámez-Guadix et al., 2023; Schittenhelm et al., 2025b). However, as they are cross-sectional, most studies do not provide insight into the stability of victimization experiences. Given the procedural nature of cybergrooming, victimization experiences may extend over a prolonged period (Van Gijn-Grosvenor & Lamb, 2021). In fact, longitudinal data revealed that 10.9% of the surveyed adolescents were stable victims over two time points one year apart (Gámez-Guadix et al., 2023). Further, using experiences of online sexual solicitation and interaction as a proxy for cybergrooming victimization, 8.3% of surveyed adolescents were stable or intermittent, i.e., re-victimized, victims over three time points spread over 13 months (Ortega-Barón et al., 2022). These findings suggest that for several young people, cybergrooming victimization does not represent a one-off or short-term incident but can be chronic or recurring. While previous studies have identified potential risk factors for the onset of cybergrooming victimization, such as socio-demographic characteristics like older age (e.g., Calvete et al., 2022), being female (e.g., Bergmann & Baier, 2016), and a non-heterosexual orientation (e.g., Gámez-Guadix et al., 2018), there is little evidence on which factors contribute to the (de)stabilization of cybergrooming victimization. However, based on an adapted version of the General Aggression Model, it can be argued that various psychosocial factors may contribute not only to the onset but also to the stability of victimization.

### Application of the General Aggression Model to Cybergrooming Victimization

**Fig. 1:**
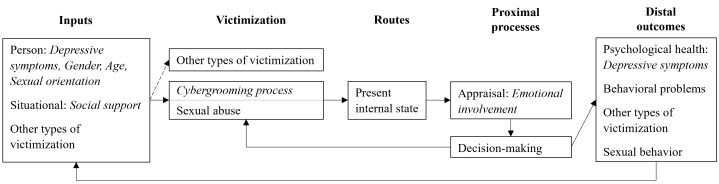
Adapted Version of the General Aggression Model. Terms in italics indicate the variables incorporated in analyses in this study. The model was adapted from Schittenhelm et al. (2025a).

Although the General Aggression Model (GAM; Anderson & Bushman, 2002) was introduced as a framework for explaining aggressive behavior, it has been extended to also address victimization experiences (e.g., Kowalski et al., 2014). It has also been adapted to describe the interplay of risk factors, victimization experiences, and their consequences in the context of cybergrooming victimization (Schittenhelm et al., 2025a). In short, according to the adapted GAM (see Fig. 1), a person’s exposure risk to cybergrooming victimization depends on the interaction among several risk factors, referred to as *inputs* (e.g., socio-demographic characteristics). Once victimized, victims experience internal states through cognitive, affective, and arousal *routes* (e.g., affection). These are fundamental to *proximal processes*, namely, the victims’ appraisal of the cybergrooming situation, based on which they make decisions (e.g., engaging in sexual interactions). *Distal outcomes* of victimization are conceptualized as adverse consequences on different levels (e.g., impaired psychological health, behavioral problems) that can feed back into risk factors. For a more detailed description of the GAM applied to cybergrooming victimization, see Schittenhelm et al., (2025a). This brief description, however, already provides explanatory approaches to the stability of cybergrooming victimization, as outlined in the adapted GAM.

First, revictimization may occur due to a feedback loop from distal outcomes to risk factors. That is, some outcomes may promote risk factors, or, alternatively, some factors can be both outcomes and risk factors. Accordingly, it has been suggested that traumatic dynamics following cybergrooming victimization may lead to increased depressive symptoms, but that depressive symptoms may also make potential victims appear more vulnerable to perpetrators (Gámez-Guadix et al., 2023). Although not yet well researched in the context of cybergrooming, there is longitudinal evidence for a bidirectional relationship between depressive symptoms and victimization, particularly regarding peer victimization (Christina et al., 2021). To summarize, cybergrooming victimization may foster depressive symptoms, which in turn increase the risk of later revictimization.

Second, proximal processes as postulated by the model can be considered in explaining the chronicity of victimization. It has been proposed that, resulting from perpetrators’ strategies to manipulate the victims’ appraisal of the situation, victims make decisions that contribute to the continuity of victimization (Schittenhelm et al., 2025a). For example, some victims may perceive the cybergrooming situation initially as an exciting experience or a genuine relationship (Whittle et al., 2015) (*appraisal*) that they wish to continue (*decision-making*). A key factor in this regard may be the victim’s emotional involvement. It can be hypothesized that the more important the “relationship” to the victim is, the less likely they are to (or can) leave the situation. Victims may be emotionally involved despite experiences of violence and abuse, which the psychological mechanism of trauma bonding can explain. This describes an emotional bond from victim to perpetrator in a relationship characterized by emotional dependency, a power differential, and intermittent abuse (McElvaney, 2019). It was pointed out that, in both offline and online contexts, the interplay of grooming strategies of positive enticement (e.g., gift-giving, compliments) and coercion (e.g., threats, blackmail) generates precursors to trauma bonding (Bilali & Reid, 2024).

Third, while depressive symptoms and emotional involvement are understood as factors that may increase the risk of chronicity or revictimization, social support may decrease it. Social support is considered a protective factor against sexual revictimization (e.g., Mason et al., 2009; Strøm et al., 2020). In the specific context of cybergrooming, initial research indicated a negative cross-sectional association between family support and victimization risk (Aktu, 2024; Pasca et al., 2022). However, the role of social support for the risk of repeated victimization has not been investigated yet. Since social support and depressive symptoms are linked in adolescence (e.g., Pössel et al., 2018), there are several conceivable pathways (e.g., an interaction of these factors on the input level or a buffering effect of social support on depressive symptoms after victimization), which may be relevant to the onset and stability of cybergrooming victimization. However, a description of the interplay between these factors is beyond the scope of the present study.

### (Lacking) Victim Typologies: A Person-Centered Approach to Victimization Experiences

Research on cybergrooming perpetrators has yielded several attempts to typologize them, for instance, based on employed strategies and motivations (e.g., Fortin et al., 2025; Kloess et al., 2019; Tener et al., 2015). Breaking down this body of research, a recent systematic review described a rough differentiation between intimacy-seeking perpetrators and sexually preoccupied perpetrators, with the former being primarily interested in an intimate relationship with the victim and the latter in the quick satisfaction of sexual needs (Moosburner et al., 2025). This differentiation is reflected in strategies employed by perpetrators. For example, the sexually preoccupied type is more likely to engage in aggressive strategies and less likely to adopt strategies aimed at relationship building than the intimacy-seeking type. Referring to victims of cybergrooming, hardly any attention has been paid to potential victim types. Only based on the behavior and reports of cybergrooming perpetrators, two types of victims have been tentatively proposed: the vulnerable type, characterized by, for example, a need for attention and affection, and the risk-taking type, characterized by, for instance, disinhibition and adventure seeking (Webster et al., 2012). The authors hypothesized that there may be a link between perpetrator and victim type, in that intimacy-seeking groomers seem more likely to match with the vulnerable victim type. In contrast, sexually preoccupied groomers seem more likely to match with the risk-taking type. Currently, there is no research on this hypothesis. However, from a theoretical perspective, distinct patterns of cybergrooming experiences may exist among victims, characterized by unique combinations of strategies they encounter. For example, there may be a group of victims of primarily sexually preoccupied perpetrators who are more likely to experience aggression than profound interest in their person. At the same time, this may be reversed for victims of primarily intimacy-seeking perpetrators, simply put.

Statistically, assumptions about the existence of groups of individuals can be investigated using person-centered approaches that group individuals based on their response behavior. Person-centered approaches have already been employed in the context of cybergrooming victimization, primarily to investigate how this type of victimization clusters with other types of victimization or perpetration, such as (cyber)bullying and offline abuse (e.g., Calvete et al., 2020; Rindestig et al., 2025). However, to date, studies focusing on cybergrooming victimization experiences have employed variable-centered approaches (i.e., examining relationships between variables). While these studies showed, for example, that the grooming strategies described above are all intercorrelated (Gámez-Guadix et al., 2021; Schittenhelm et al., 2025b), they did not provide any information on whether relevant victim subgroups emerge among young people. Person-centered approaches represent a valuable tool for addressing this research gap. As evident in research on other types of victimization, such as peer victimization in early adolescence (Nylund et al., 2007b) and cybercrime victimization among adults (Lee & Wang, 2024), such analyses may result in groups that primarily demonstrate *quantitative* differences. That is, in the context of the present study, different groups would exhibit similar patterns of experienced grooming strategies but differ in the likelihood of experiencing them. Alternatively, building on the suggestion that there could be a match between perpetrator types and potential victim types (Webster et al., 2012), person-centered approaches may reveal groups that exhibit *qualitative* differences in their experiences of victimization. That is, groups would exhibit distinct patterns of experienced strategies.

## The Present Study

Cybergrooming represents a common online threat affecting young people. Although online sexual victimization in general and cybergrooming victimization in particular have gained scientific attention, several research questions remain unanswered. Research focusing on perpetrators has demonstrated that they can be organized into meaningful subgroups, which differ, for example, in the strategies they employ. However, no research has been conducted yet on the identification of victim groups according to the strategies they encounter. Therefore, the first objective of the present study was to examine whether latent classes of adolescent cybergrooming victims can be identified based on their experienced grooming strategies, and if so, how these classes differ qualitatively and/or quantitatively. Further, given the occurrence of stable victimization trajectories, the second objective was to investigate the stability of, or changes in, victimization across time. Based on considerations derived from an adaptation of the GAM, this study examined the effects of selected socio-demographic and psychosocial factors on transitions in class membership over time, considering how these factors may be involved in the onset and stability of victimization.

## Methods

### Sample and Data Cleaning

The initial sample consisted of 1937 Spanish adolescents. Participants were excluded if they had only missing values on at least one construct of interest at T1 or T2 (*n* = 1057; mainly due to dropout to T2) or showed highly consistent response behavior at T1 or T2 (*n* = 192; i.e., choosing the same response option within each construct of interest). Thus, the final sample size was *N* = 688. The mean age of the final sample at T1 was 13.86 years (*SD* = 1.22, range: 12–18). 56% of participants identified as female, 41.4% as male, and 1.3% as non-binary; 1.3% did not report their gender. Overall, 88.5% of the sample identified as heterosexual, 2.8% as homosexual, 6.5% as bisexual, and 1.6% reported having another sexual orientation; 0.5% did not report their sexual orientation.

### Procedure

The study was reviewed and approved by the Autonomous University of Madrid Ethics Committee. The present study is part of a larger project on online (sexual) victimization of youth; see Gámez-Guadix et al. (2023) for a detailed description of the procedure.

### Measures

All items analyzed in the present study are provided in English translation from Spanish in Table S1 of the supplementary material.

#### Cybergrooming victimization 

Cybergrooming victimization was assessed at both time points with the Multidimensional Online Grooming Questionnaire (MOGQ; Gámez-Guadix et al., 2021), comprising five subscales reflecting common cybergrooming strategies, namely deception (e.g., “[…] deceived me into believing we had things in common or liked the same things”), gift-giving (e.g., “[…] offered me money or other things in exchange for photos or videos of myself”), interest in the victim environment (e.g., “[…] showed interest in my school schedules, family, etc.”), sexualization (e.g., “[…] asked me about my sexual experiences”), and aggression (e.g., “[…] sent me threatening or insulting messages”). All 20 items referred to the past 12 months and to perpetrators whom the participants believed to be adults and suspected of having sexual intentions toward them. They could be answered on a 4-point Likert scale ranging from “Never” (0) to “5 times or more” (3).

#### Depressive symptoms 

Depressive symptoms were assessed at both time points with the respective subscale of the Brief Symptom Inventory (Derogatis & Fitzpatrick, 2004). All six items (e.g., “Feeling sad”, “Losing interest in things”) referred to the past two weeks and could be answered on a 5-point Likert scale ranging from “Not at all” (1) to “A lot” (5).

#### Social support 

Social support, as a measure of resilience, was assessed at T1 using the respective subscale of the Resilience Scale for Adolescents (Ruvalcaba-Romero et al., 2015). All four items (e.g., “I have some friends and family members who really care about me.”) could be answered on a 5-point Likert scale ranging from “Strongly disagree” (0) to “Strongly agree” (4).

#### Emotional involvement 

Emotional involvement was measured with four items (e.g., “I was so busy with our communication that I had little time for other things.”), which could be answered on a 4-point Likert scale ranging from “Never” (0) to “5 times or more” (3).

### Analytical Approach

Data inspection and preparation, descriptive analyses, and factor analyses were conducted with R (R Core Team, 2025). Latent class analysis (LCA) and latent transition analysis (LTA) were conducted with Mplus (Muthén & Muthén, 2024), as it is more convenient than R for applying the BCH method in LTA (see below). To make all analyses reproducible, all materials necessary are provided in an online repository: https://osf.io/ej53n/files/osfstorage.

#### Factor analyses 

To test the dimensionality of the constructs of interest and justify the computation of manifest means across items, confirmatory factor analyses (CFAs) were performed using the R package *lavaan* (Rosseel, 2012). Maximum likelihood with robust standard errors (MLR) was used for estimation, with missing data being handled using full information maximum likelihood. Robust Comparative Fit Index (CFI), robust Root Mean Square Error of Approximation (RMSEA), and Standardized Root Mean Square Residual (SRMR) were considered as fit indices to evaluate model fit. Good model fit was indicated by CFI ≥ 0.95, RMSEA ≤ 0.06, and SRMR ≤ 0.08 (Hu & Bentler, 1999). Acceptable model fit was indicated by CFI ≥ 0.90, RMSEA ≤ 0.08, and the SRMR ≤ 0.10 (Bentler, 1990; Browne & Cudeck, 1992). For scale reliability estimation, McDonald’s omega (ω; McDonald, 1999) was used, with ω ≥ 0.65 indicating acceptable reliability (Kalkbrenner, 2023).

To ensure comparability of latent classes at T1 and T2, longitudinal measurement invariance of cybergrooming victimization, as measured by the MOGQ, was examined. Configural invariance (i.e., equal model structure), metric invariance (i.e., equal loadings), and scalar invariance (i.e., equal intercepts) were tested consecutively. Following established recommendations, ΔCFI ≥ − 0.010, ΔRMSEA ≥ 0.015, and ΔSRMR ≥ 0.030 indicated a lack of metric invariance, and ΔCFI ≥ − 0.010, ΔRMSEA ≥ 0.015, and ΔSRMR ≥ 0.010 indicated a lack of scalar invariance (Chen, 2007).

#### Latent class and transition analyses 

A common person-centered approach is LCA, which probabilistically classifies individuals into unobserved classes based on their response behavior to categorical indicators (Bauer, 2022). LTA is an extension of LCA, which allows for examining both research objectives jointly, namely (1) the identification of distinct victim groups and (2) the analysis of the stability of cybergrooming victimization. LTA goes beyond merely identifying latent classes at different time points to examine stability and change in categorical states across time (Nylund-Gibson et al., 2023). Importantly, LTA enables the analysis of the effects of covariates on transitions between classes (Asparouhov & Muthén, 2021).

First, to determine which class solutions (i.e., the number of classes) to use in LTA, LCAs with different class solutions were estimated separately for each time point. Mean values across the respective items of each cybergrooming subscale were calculated to reduce model complexity and then dichotomized (1 = mean value greater than 0; 0 = mean value equals 0), indicating whether a particular cybergrooming strategy had been experienced. The dichotomized means served as indicators in LCA. The dichotomization of mean values was carried out for LCA because a latent profile analysis (LPA), a comparable person-centered approach classifying persons probabilistically into unobserved profiles based on continuous indicators, with the mean values as indicators, did not yield substantive and informative profiles. This was probably due to the strongly right-skewed distribution of the MOGQ items, since only a few participants experienced the described situations frequently. Models with one to four classes were estimated using the robust maximum likelihood estimator (MLR). To avoid local optima, all LCAs were estimated with 5000 random starting values, retaining the 200 best solutions for final optimization over 1000 iterations. For model selection, it is recommended to consider both statistical criteria and the theoretical interpretability of each class solution (Weller et al., 2020). Therefore, class solutions were compared using bootstrapped Likelihood Ratio tests (BLRT), Akaike information criterion (AIC), and sample-size-adjusted Bayesian information criterion (saBIC) as information criteria (Nylund et al., 2007a), as well as the interpretability of classes. Because LCA uses categorical indicators, the resulting classes were interpreted and compared using the estimated conditional item probabilities, which represent the probability of particular responses to indicators (i.e., whether someone experienced a grooming strategy) for each class (Lanza & Cooper, 2016). As diagnostic criteria, entropy and the size of the smallest class were considered. Entropy is a measure of classification accuracy, with values ≥ 0.80 considered desirable (Bauer, 2022). As a rough guideline, the smallest class should not comprise less than 5% of the sample, but above all, it should make conceptual sense (Weller et al., 2020). Additionally, after model selection, classes of the selected model were compared with respect to demographic and psychosocial variables. To this end, the BCH method (Asparouhov & Muthén, 2021) was applied, which estimates the mean values of specified auxiliary variables within classes while accounting for classification error. This is necessary because assigning individuals to classes involves uncertainty and treating class membership as deterministic may lead to biased results (Bauer, 2022). Differences in BCH estimates of mean values were tested for significance based on Χ^2^-tests.

LTA was then conducted using a multi-step estimation procedure, likewise, based on the BCH method (Asparouhov & Muthén, 2021). First, LCAs were estimated using the previously determined class solutions; however, this time in a joint model to estimate and save joint BCH weights that reflect the measurement error of the latent class variables. To ensure a consistent ordering of classes across time points (e.g., class 1 denotes the same class at both time points) and thus easier interpretability, the starting values for the class thresholds were defined in the input file based on prior model outputs from separate LCAs. In the next step, the latent transition model was estimated based on the joint BCH weights. In the final step, demographic and psychosocial variables were added to the model to examine their effect on transitions. Only T1 variables, i.e., variables that precede transitions in time, were considered in these analyses to examine which variables predict transitions.

## Results

### Factor Analyses

Prior to the main analyses, CFAs were conducted to examine the dimensionality of each construct and to determine which items to retain for the following analyses. For cybergrooming victimization, a correlated factors model was examined, and for depressive symptoms, social support, and emotional involvement, g-factor models were examined. Detailed model fits for all tested models are provided in Table S2 of the supplementary material. The correlated factors model for cybergrooming victimization exhibited acceptable model fit with all initial items at T1 and T2. However, two gift-giving items had very low loadings especially at T1 (λ_1T1_ = 0.30, λ_2T1_ = 0.26; impacting ω of gift-giving at T1 with ω_1_ = 0.63) and one aggression item had a non-significant loading at T2 (λ_2T2_ = 0.19). For congruency of subscales at both time points, these items were excluded from further analyses for T1 and T2. The final model exhibited good model fit at both time points. One item on depressive symptoms had to be excluded at both time points for the unidimensional model to achieve acceptable-to-good model fit. A unidimensional model for social support indicated good model fit with all initial items. The unidimensional models for emotional involvement were based on the subsample of victims at each time point (i.e., participants who had experienced at least one of the cybergrooming situations). At T2, one item had to be excluded for the unidimensional model to reach good model fit. Therefore, the corresponding item was also excluded from the model for emotional involvement at T1, also demonstrating good model fit. Since all final models indicated at least an acceptable model fit, the calculation of manifest means across the remaining items for the following analyses was justified.

Measurement invariance of cybergrooming victimization was first examined separately for each subscale and subsequently for the correlated factors model. For both the subscales and the correlated factors model, configural invariance model fit and changes in fit indices from metric to scalar invariance models supported measurement invariance. For simplicity, Table 1 depicts results only for the correlated factors model (see Table S3 of the supplementary material for invariance models per subscale). Therefore, comparing cybergrooming victimization as measured by the MOGQ at T1 and T2 was considered meaningful.

**Table 1:** Measurement invariance models for the correlated factors model

Model	χ^2^ (df)	rCFI	rRMSEA	SRMR	Δ rCFI	Δ rRMSEA	ΔSRMR
Configural	747.70 (465)	0.953	0.046	0.052			
Metric	731.06 (477)	0.955	0.044	0.055	0.002	− 0.002	0.003
Scalar	772.86 (495)	0.950	0.046	0.059	− 0.005	0.002	0.004

Note. rCFI = robust CFI, rRMSEA = robust RMSEA, Δ = difference in robust CFI/robust RMSEA/SRMR values of nested models

### Preliminary Analyses

In the total sample, 30.1% of participants (*n* = 207) were T1-victims, and 31.5% of participants at T2 (*n* = 217) were T2-victims. Of these, 120 participants, i.e., 17.4% of the total sample, were victims at both T1 and T2, and were thus considered stable victims across the two time points. Table 2 presents the mean values across subscales for the total sample, as well as the number of participants who experienced at least one instance of the respective strategy. Descriptive statistics for all single items can be found in Table S1 of the supplementary material. Although the number of victims remained relatively stable over time, most individual strategies were experienced more often at T2 than at T1. This indicates that more participants experienced multiple strategies at T2 than at T1. Depressive symptoms increased slightly on average over time (*M*_1_ = 2.09, *SD*_1_ = 0.93; *M*_2_ = 2.39, *SD*_2_ = 1.02; *t*(687) = -7.94, *p* < .001). Participants reported having a rather high level of social support (*M* = 3.33, *SD* = 0.83). Descriptively, T1-victims and T2-victims indicated a comparable mean level of emotional involvement (*M*_1_ = 0.27, *SD*_1_ = 0.55; *M*_2_ = 0.21, *SD*_2_ = 0.43). Manifest correlations between single items are presented in Table S4 of the supplementary material.

**Table 2:** Descriptive statistics for MOGQ subscales

	Deception		Gift giving		Interest		Sexualization		Aggression	
	T1	T2	T1	T2	T1	T2	T1	T2	T1	T2
*M* (*SD*)	0.12 (0.36)	0.14 (0.34)	0.05 (0.32)	0.07 (0.32)	0.20 (0.52)	0.18 (0.45)	0.12 (0.42)	0.16 (0.46)	0.06 (0.27)	0.07 (0.28)
∑ (%)	123 (17.9)	145 (21.1)	30 (4.4)	41 (6.0)	148 (21.5)	144 (20.9)	92 (13.4)	112 (16.3)	54 (7.8)	61 (8.9)

Note. ∑ = Number and percentage of participants who experienced at least one situation of the respective subscale at least once

### Latent Class Analysis

Table 3 depicts model fit and diagnostic criteria for LCA models with one, two, three, and four classes. Information criteria (IC) were smallest for the three-class solution at both time points. Furthermore, when a fourth class was added, the BLRT was no longer significant at both T1 and T2. However, since IC for the four-class solution were similarly low, interpretability of the three- and four-class solutions were compared. Adding a fourth class led to the emergence of a very small, highly specific class for both T1 and T2, but the classes were not comparable in content (i.e., regarding the strategies experienced in this class). Additionally, at both T1 and T2, one class in the three-class solution almost perfectly represented those who had never experienced any of the cybergrooming situations, whereas in the four-class solutions, several victims and non-victims were mixed into a single class. Therefore, the three-class solution was selected as the final model. At both time points, diagnostic criteria for this solution were satisfying as entropy was at least 0.80 and the smallest class contained at least 5% of the sample.

**Table 3:** Evaluation of LCA solutions

Model	AIC	saBIC	BLRT	Entropy	Smallest class
**T1**					
1 class	2538.94	2545.73		1.00	1.00
2 classes	2028.30	2043.25	< 0.0001	0.86	0.19
3 classes	1999.38	2022.48	< 0.0001	0.83	0.05
4 classes	2005.63	2036.87	1.00	0.88	0.01
**T2**					
1 class	2758.61	2765.40		1.00	1.00
2 classes	2139.65	2154.60	< 0.0001	0.88	0.21
3 classes	2128.18	2151.28	< 0.0001	0.80	0.11
4 classes	2131.69	2162.94	0.286	0.86	0.02

Note. The smallest class is given as a percentage of the total sample

**Fig. 2:**
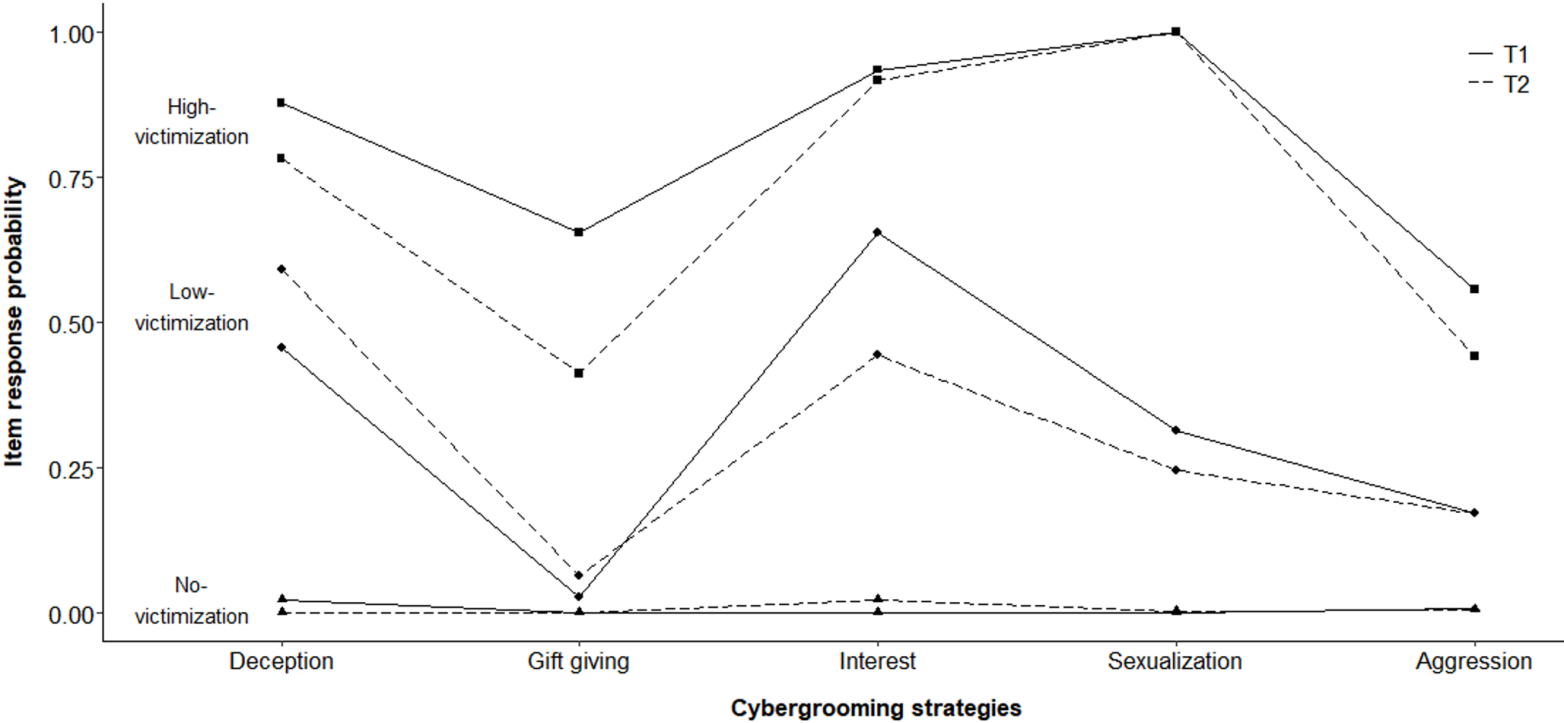
Conditional Item Response Probabilities for Each Class at T1 and T2

At T1, one class almost perfectly represented the non-victims (i.e., all participants, who had not experienced any of the cybergrooming situations). There was only one participant assigned to the corresponding class, who experienced gift-giving (but no other strategy). At T2, the respective class perfectly represented the non-victims. These classes were designated as the no-victimization class at both time points for simplicity, as only one single victim was classified into the respective class at T1. The other two classes were designated as the low- and high-victimization classes. Item response probabilities of all classes were comparable across both time points, except that gift-giving was less likely in the high-victimization class at T2 and interest was less likely in the low-victimization class at T2 (see Fig. 2). At both time points, item response probabilities were higher for each cybergrooming strategy in the high-victimization class than in the low-victimization class. A more detailed analysis based on most likely class membership showed that members of the high-victimization classes were very likely to experience multiple strategies (especially sexualization, interest, and deception). In contrast, members of the low-victimization class predominantly experienced only one to two strategies (see Table 4). According to the most likely class membership, 36 participants were assigned to the high-victimization class at T1, 170 to the low-victimization class, and 482 to the no-victimization class. At T2, 77 participants were assigned to the high-victimization class, 140 to the low-victimization class, and 471 to the no-victimization class. This enlargement of the high-victimization class was particularly attributable to the fact that at T2, more victims who had experienced three strategies were assigned to the high-victimization class than at T1 (see Table 4). That is, there was a slight inconsistency in classification regarding those experiencing three strategies. Essentially, this inconsistency stemmed from the fact that some patterns (particularly the combination of deception, interest, and sexualization) were assigned to the low-victimization class at T1 but to the high-victimization class at T2. A detailed description of this inconsistency is provided in the supplementary material.

**Table 4:** Number of experienced strategies within classes based on most likely class membership

Class	Number of experienced strategies				
	1	2	3	4	5
Low-victimization T1	74	68	28	-	-
Low-victimization T2	71	59	10	-	-
High-victimization T1	-	-	4	20	12
High-victimization T2	-	-	36	29	12

Acknowledging that labeling classes as low- and high-victimization implies a difference in the severity of victimization beyond the number of strategies experienced, it was examined whether experiencing multiple strategies was also associated with experiencing the respective individual strategies more frequently. The supplementary material (page 7) contains a detailed elaboration on why the present data support this assumption, concluding that the designation of low- and high-victimization is appropriate.

Subsequently, classes were compared on demographic and psychosocial variables using the BCH method to test for differences across classes while accounting for classification uncertainty. As the number of sexual minority youth was rather small, sexual orientation had to be dichotomized to be included in the analyses (0 = heterosexual, 1 = homosexual, bisexual, or another sexual orientation). Furthermore, only female (coded as 0) and male (coded as 1) participants were considered in the analyses on gender effects, as the number of non-binary participants was too small (*n* = 9). Table 5 presents the BCH estimates of means for relevant variables across classes, indicating significant differences between classes. First, results showed that being in the high-victimization class (and low-victimization class at T1) was associated with higher age. Further, sexual minority orientation was more common in the high-victimization than in the no-victimization class at T1 but not at T2. However, this could be related to the fact that at T1 and T2, those who had experienced three strategies were classified inconsistently. More precisely, almost all individuals classified into the high-victimization class at T2 who experienced three strategies were heterosexual, which suppressed the overrepresentation of sexual minority youth in this class in contrast to T1. At both time points, being a girl was more common in the high-victimization than in the no- and low-victimization classes. When examining the T1-classes, all classes differed in depressive symptoms at T1 and T2, with the no-victimization class exhibiting the lowest and the high-victimization class exhibiting the strongest symptoms. A similar result was evident for the T2-classes, except that the no- and low-victimization classes did not differ in terms of their T1 depressive symptoms. Descriptively, the no-victimization class scored highest on social support, but none of the pairwise differences reached significance. Finally, both at T1 and T2, the low-victimization class was less emotionally involved than the high-victimization class.

**Table 5:** Parameter estimates with standard errors for each class

	Gender*	Age T1	Sexual orientation*	Depressive symptoms T1	Depressive symptoms T2	Social support T1	Emotional involvement T1	Emotional involvement T2
**T1-classes**								
No-victimization	0.47 (0.02)	13.68 (0.06)	0.09 (0.01)	1.95 (0.04)	2.27 (0.05)	3.38 (0.04)	-	-
Low-victimization	0.37 (0.04)	14.15 (0.12)	0.11 (0.03)	2.26 (0.09)	2.53 (0.09)	3.24 (0.08)	0.18 (0.04)	-
High-victimization	0.15 (0.08)	14.80 (0.21)	0.30 (0.09)	3.01 (0.20)	3.18 (0.21)	3.15 (0.18)	0.76 (0.15)	-
Significant differences	N, L < H	N < L < H	L < H	N < L < H	N < L < H		L < H	
**T2-classes**								
No-victimization	0.47 (0.02)	13.75 (0.06)	0.10 (0.02)	1.97 (0.04)	2.24 (0.04)	3.39 (0.04)	-	-
Low-victimization	0.43 (0.05)	13.77 (0.13)	0.12 (0.04)	2.12 (0.10)	2.51 (0.12)	3.18 (0.10)	-	0.09 (0.03)
High-victimization	0.15 (0.06)	14.71 (0.17)	0.16 (0.05)	2.77 (0.15)	3.08 (0.17)	3.25 (0.11)	-	0.45 (0.08)
Significant differences	N, L < H	N, L < H		N, L < H	N < L < H			L < H

Note. N = no-victimization, L = low-victimization, H = high-victimization. *With the BCH method, binary auxiliary variables are treated as continuous, which is a valid method when the binary variable is coded 0/1 (Asparouhov & Muthén, 2021)

### Latent Transition Analysis

**Fig. 3:**
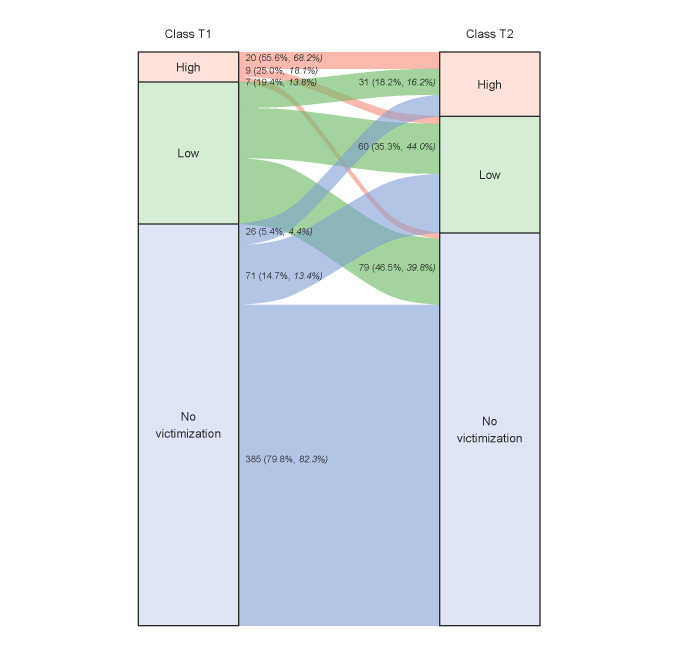
Latent Transition Probabilities Based on Most Likely Class Membership and BCH Method. Note. The illustrated transitions are based on the most likely class membership; percentages in italics refer to transition probabilities derived from LTA using the BCH method

When examining transitions based on the most likely class membership, results showed that most participants assigned to the no-victimization class at T1 remained in it at T2. However, some members transitioned to the low-victimization class and even to the high-victimization class. Almost half of the low-victimization class members transitioned to the no-victimization class, while about one-third remained in the low-victimization class. Noticeably, those in the low-victimization class who experienced sexualization were more likely to remain in this class or transition to the high-victimization class than those who did not experience sexualization. More than half of the participants assigned to the high-victimization class at T1 remained in this class at T2, while approximately the same number transitioned to the no- and low-victimization classes. Figure 3 illustrates these results. Comparing these figures with the LTA results with the BCH method revealed a few deviations (especially greater stability in the low- and high-victimization classes), but the overall pattern was very similar. Thus, the pattern of transition probabilities was not substantially affected by accounting for classification uncertainty, as expected given the desirable entropy of class solutions at both time points.

Table 6 illustrates the impact of selected socio-demographic and psychosocial factors on the odds of transition. LTA clearly indicated that age is a risk factor for problematic transitions. That is, the odds of transitioning from no- or low-victimization to high-victimization increased by 41% and 65%, respectively, with each additional year. Congruently, the odds of transitioning from high-victimization to no- and low-victimization decreased by 29% and 39% per year, respectively. Being a boy decreased the odds of transitioning from the no-victimization to the high-victimization class by 69% and increased the odds of transitioning from the high-victimization to the no-victimization class by 219%. Descriptively, further odds ratios by gender were notable (e.g., OR = 2.96 for the transition from the high-victimization to the low-victimization class), but these were not statistically significant due to wide 95% confidence intervals. Furthermore, the findings showed that the odds of transitioning from the no-victimization to the high-victimization class increased by 56%, while the odds of transitioning from high-victimization to no-victimization decreased by 36% per one-point increase in depressive symptoms on the Likert scale. Finally, one Likert point on emotional involvement decreased the odds for transitioning from high-victimization to no-victimization by 76%. Descriptively, emotional involvement influenced transition odds from low- to high-victimization (OR = 3.37) and from high- to low-victimization (OR = 0.30); however, these effects did not reach significance due to wide 95% confidence intervals. Social support and sexual orientation did not have a significant effect on transition odds.

**Table 6:** Transition Odds Ratios With 95% Confidence Intervals

Class T1	Predictor	Class T2		
		No-victimization	Low-victimization	High-victimization
No-victimization	Gender	-	0.93 [0.52; 1.65]	**0.31** [0.11; 0.93]
	Age	-	0.86 [0.66; 1.11]	**1.41** [1.01; 1.98]
	Sexual orientation	-	0.76 [0.25; 2.70]	0.94 [0.27; 3.48]
	Depressive symptoms	-	1.07 [0.77; 1.48]	**1.56** [1.06; 2.28]
	Social support	-	0.81 [0.58; 1.14]	1.07 [0.70; 1.71]
Low-victimization	Gender	1.08 [0.60; 1.92]	-	0.34 [0.10; 1.17]
	Age	1.17 [0.90; 1.51]	-	**1.65** [1.09; 2.49]
	Sexual orientation	1.32 [0.44; 3.95]	-	1.27 [0.24; 6.64]
	Depressive symptoms	0.94 [0.68; 1.29]	-	1.46 [0.90; 2.35]
	Social support	1.23 [0.88; 1.73]	-	1.32 [0.75; 2.33]
	Emotional involvement	0.82 [0.18; 3.78]	-	3.37 [0.99; 11.47]
High-victimization	Gender	**3.19** [1.08; 9.46]	2.96 [0.85; 10.24]	-
	Age	**0.71** [0.51; 0.99]	**0.61** [0.40; 0.92]	-
	Sexual orientation	1.04 [0.29; 3.74]	0.79 [0.15; 4.10]	-
	Depressive symptoms	**0.64** [0.44; 0.94]	0.69 [0.43; 1.11]	-
	Social support	0.94 [0.59; 1.49]	0.76 [0.43; 1.34]	-
	Emotional involvement	**0.24** [0.07; 0.84]	0.30 [0.09; 1.01]	-

## Discussion

Despite evidence of stable cybergrooming victimization experiences, factors that influence stability and changes in victimization status over time have hardly been addressed previously. In addition, prior studies on cybergrooming have only successfully identified perpetrator typologies, while efforts to identify victim typologies are lacking. Addressing these research gaps, the present study examined cybergrooming victimization among Spanish adolescents across time by means of LTA. LCA yielded three classes at both time points (no-victimization, low-victimization, and high-victimization classes), which differed (a) primarily quantitatively but not qualitatively and (b) on selected socio-demographic (e.g., gender) and psychosocial variables (e.g., depressive symptoms). LTA showed high stability across the no- and high-victimization classes, and that transitions between these classes were influenced by several of the selected socio-demographic and psychosocial variables.

### Lacking Victim Typology

In LCA with binary indicators coding whether the respective cybergrooming strategy was experienced, the three-class solution exhibited the best fit at both time points. At both time points, a no-victimization class emerged, in which the probability of experiencing a given strategy was (almost) zero. These classes represented the non-victims almost perfectly and thus constituted the largest classes. This finding joins previous research on different types of victimization (and perpetration) among young people using person-centered approaches. For example, studies focusing on (cyber)bullying (Antoniadou et al., 2019), peer sexual coercion (French et al., 2014), and interpersonal violence (e.g., sexual assault, dating violence; Sessarego et al., 2021) each yielded one group that was not involved and the largest one. This supports the appropriateness of the three-class solution in the present LCA. Beyond that, two victim subgroups were identified, namely a low- and a high-victimization class. At both time points, these two classes differed mainly quantitatively rather than qualitatively, as they exhibited a similar pattern of experienced strategies. That is, deception and interest were experienced more often than gift-giving and aggression in both classes. Congruently, gift-giving and aggression were likewise the least frequently experienced strategies in a sample of German adolescent victims (Schittenhelm et al., 2025b). This mainly quantitative distinction between classes was also reflected in the fact that (when examining classes based on the most likely class membership) members of the high-victimization class have experienced multiple strategies, while in the low-victimization class, only one or two strategies were predominantly experienced. Further analyses also indicated that experiencing multiple strategies is associated with experiencing them more frequently. However, the classification of those who experienced three strategies did not fully match across time points. Still, the classes can mainly be characterized according to differences in the amount or frequency of experienced strategies, rather than the pattern of these strategies. Thus, overall, while research on perpetrators suggests qualitatively different types of perpetrators (Moosburner et al., 2025), the present findings did not reveal qualitatively different types of victims, who, as theoretically considered, experience strategy patterns in accordance with the respective perpetrator type to which they fall victim.

### Stability of Victimization Experiences

The only qualitative difference between the low- and high-victimization classes was that everyone in the high-victimization class experienced sexualization, whereas in the low-victimization class, it was only the third most likely strategy. This difference can be discussed in relation to the moderate stability of the low-victimization class. The composition of the low-victimization class at T1 showed that it includes (1) victims who were able to escape the cybergrooming situation, i.e., who transitioned to no-victimization, and (2) victims for whom low-victimization was stable or represented a precursor to high-victimization. Interestingly, experiencing sexualization seems to play a role here, as experiencing sexualization was associated with staying in the same class or transitioning to the high-victimization class when analyzing transitions based on most likely class membership. Although some perpetrators use sexualization very quickly (Kloess et al., 2019), it could be argued that especially the strategies of deception and interest in the victim’s environment are often used in the beginning of the grooming process to establish contact with the victim and build a connection, on the basis of which sexual content is to be introduced (Santisteban et al., 2018). For several members of the low-victimization class, the grooming process may have been terminated before sexualization began because, for example, they may have become suspicious. The fact that the high-victimization class, in which every member experienced sexualization, showed high stability is consistent with the reasoning that sexualization may be decisive for victimization stability.

While it is encouraging that most non-victims remained non-victims, it is concerning that stability was this high in the high-victimization class. In addition, some high-victimization class members transitioned to the low-victimization class and, thus, still remained victimized. It is crucial to understand what distinguishes less vulnerable adolescents from high-victims and stable low-victims experiencing sexualization to identify factors that prevent progression or stability of low and high victimization. Or, in other words: Why are some adolescents particularly resilient (i.e., especially stable non-victims) and others particularly vulnerable (i.e., especially stable members of the high-victimization class and members of the low-victimization class transitioning to the high-victimization class)? While most socio-demographic and psychosocial factors examined in the present study did not significantly influence transitions from the low-victimization class to other classes, several of them predicted transitions between the no- and high-victimization classes. The fact that few effects were observed for transitions between low- and high-victimization classes could partly be attributed to the inconsistent classification of participants across three strategies, which slightly blurred these classes over time.

#### Role of socio-demographic factors 

The findings of the present study indicate that all examined socio-demographic factors are relevant to cybergrooming victimization experiences. First, girls were more likely to transition from the no-victimization to the high-victimization class, which is consistent with previous research suggesting that being female is a risk factor for victimization (e.g., Bergmann & Baier, 2016; Machimbarrena et al., 2018). Furthermore, the gender imbalance was considerably higher in the high-victimization class compared to the other classes, and girls were particularly vulnerable to remaining in the high-victimization class. Thus, being female can be considered a risk factor for experiencing multiple grooming strategies and high-victimization stability. A previous study indicated that perpetrators approach female victims differently than male victims, namely in that more effort is put into rapport building with girls, while sexual topics are introduced less explicitly and more carefully (Van Gijn-Grosvenor & Lamb, 2021). Reasonably, this may prolong the grooming process, but it may also strengthen the bond between the victim and the perpetrator, reducing the likelihood of detection and potentially stabilizing the victimization experience. Still, the gender composition in the no- and low-victimization classes did not significantly differ, and gender was not relevant to transitions between these classes. Thus, boys should not be neglected as potential victims.

Second, members of higher victimization classes were, on average, older than members of lower victimization classes. Further, the risk of transitioning from no- to high-victimization increased with age. Thus, age may represent a risk factor for the onset of victimization, which is in line with previous research indicating that being older constitutes a risk factor for cybergrooming victimization (Calvete et al., 2022; Gandolfi et al., 2021). Furthermore, the findings suggested that age is also relevant to the stability of victimization. With age, the risk of transitioning from the low-victimization to the high-victimization class increased, and the chance of transitioning from the high-victimization to the no- or low-victimization classes decreased. This indicates that older adolescents are more vulnerable to victimization stability. A contributing factor could be that interest in romantic relationships and sexual activity manifests itself particularly in middle and late adolescence (e.g., Gonzalez Avilés et al., 2021; Lindberg et al., 2021). Thus, older adolescents may have a greater interest in ongoing sexualized online contact, which facilitates the grooming process and its continuation for perpetrators. Specifically, willingness to engage in online sexual activity, such as sexting, likewise increases with age (Madigan et al., 2018), and sexually explicit material can be used by perpetrators to consolidate sexually exploitative relationships.

Third, the findings showed that the composition of sexual orientation differed between classes at T1. More precisely, the high-victimization class comprised more sexual minority youth than the other classes. Consistently, belonging to a sexual minority is also considered a risk factor in other types of victimization, such as sexual assault (Coulter et al., 2017). In relation to cybergrooming victimization, the higher vulnerability of sexual minority youth could be attributable to, for example, differences in Internet usage behavior. For instance, previous research showed that adolescents belonging to a sexual minority search more frequently for sexual health information online due to a lack of alternatives (Mitchell et al., 2014) and are more likely to engage in sexting than heterosexual adolescents (van Ouytsel et al., 2019). Still, at T2, classes did not differ significantly regarding their members’ sexual orientation, and sexual orientation did not significantly influence transition odds. However, the effects of sexual orientation may have been masked by the inconsistent classification of those who had experienced three strategies.

#### Role of psychosocial factors 

First, classes differed significantly in their depressive symptoms at both time points. For example, both high-victimization classes exhibited the highest depressive symptoms at both T1 and T2. This initially suggests a bidirectional relationship between depressive symptoms and victimization. However, it should be noted that the T2-variables were not controlled for by the respective T1-variables (e.g., differences in T2-depressive symptoms by T1-classes might not be that pronounced when controlling for T1-depressive symptoms, as these were also associated with T1-classes). However, building on research on other victimization types like peer victimization (Burke et al., 2017) and sexual assault (Krahé & Berger, 2017), a bidirectional relationship is conceivable in the context of cybergrooming too. As T1-depressive symptoms increased the risk of transitioning from no- to high-victimization, depressive symptoms may in fact act as a risk factor for victimization. Moreover, they decreased the chance to transition from high- to no-victimization and, thus, contributed to the stability of victimization. Based on the suggestion that depression may be both a risk factor and an outcome, as illustrated in the adapted GAM, this could be attributable to processes of revictimization. On the other hand, depressive symptoms could contribute to the continuity of victimization. For example, depressive symptoms are linked to decreased self-efficacy (Tak et al., 2017) and increased hopelessness in adolescence (Liu et al., 2021), which could impair the victim’s ability to disengage from the cybergrooming situation actively.

Second, the findings showed that although the overall level was rather low, some cybergrooming victims were emotionally involved, especially in the high-victimization class. Emotional involvement in the high-victimization class reduced the chance of transitioning to the no-victimization class, which is in line with the theoretical consideration based on the adapted GAM that emotional involvement contributes to victimization stability. Descriptively, emotional involvement also substantially increased the risk of transitioning from the low- to the high-victimization class, which, however, failed to reach significance. Thus, cybergrooming victimization experiences can be accompanied or shaped by an emotional bond experienced by the victim, raising the question as to why some victims become emotionally involved. Correlational analyses showed that, especially at T1, the strategies of sexualization and interest were most strongly associated with emotional involvement. However, it is unclear whether a certain degree of emotional involvement is a prerequisite for experiencing sexualization or whether sexualization generates emotional involvement (or both). The same applies to depressive symptoms, which were correlated with emotional involvement at T1. Overall, however, this suggests that emotional involvement interacts with the perpetrator’s strategies and the victim’s well-being.

Classes did not differ significantly in terms of social support, and it did not have a significant effect on any of the transitions. First, it is worth noting that social support was generally high in the present sample. Only a few participants reported receiving little social support from family and friends. From a statistical perspective, potential effects of social support may not have been detected due to the strongly skewed distribution. However, it is also possible that lacking social support, as measured in this study, does not in fact constitute a risk factor for the onset or stability of victimization. Congruently, neither parental nor friendship support was associated with peer victimization among adolescents (Burke et al., 2017). One explanation for the insignificant role of social support for cybergrooming experiences, as evident in the present study, could be that general social support was examined rather than social support directly related to victimization experiences and online activities. For example, social support after disclosure by victims (e.g., emotional support in coping with victimization or support in reporting the incident to authorities; Manay & Collin-Vézina, 2021) could be more relevant to destabilizing victimization experiences than general support.

### Practical Implications

First, the findings highlight vulnerable groups that should be particularly targeted by prevention and intervention efforts, especially older adolescents and girls. The best time to implement prevention programs cannot be generalized across all types of victimization. For example, in the context of anti-bullying programs, a meta-analysis showed that effectiveness decreases with age (Yeager et al., 2015). In contrast, a meta-analysis on prevention programs targeting dating violence among adolescents indicated a higher effectiveness among older adolescents (Piolanti & Foran, 2022). Since the present study demonstrated a high vulnerability to the onset and stability of victimization among older adolescents, they should be explicitly targeted by prevention and intervention efforts targeting cybergrooming victimization. One strategy could be to implement media literacy programs that address risky online behavior in general (e.g., sharing information online, expanding social networks) at a young age, while complementary programs specifically targeting sexual victimization may be more beneficial at a later stage of development. Although sexual minority orientation was more common in the high-victimization than in the no-victimization class only at T1, it should be ensured that people who deliver programs targeting sexual victimization are sensitized to sexual diversity among adolescents and acknowledge the needs and experiences of LGBTQ+ youth. Previous research emphasizes that LGBTQ+ youth are not sufficiently considered in prevention and intervention programs, even though they are at higher risk for various types of victimization (e.g., Adhia et al., 2024; Coulter & Gartner, 2023).

Second, the present findings have practical implications for the design of prevention and intervention efforts. An evaluation study of a growth mindset and self-affirmation intervention targeting online risks, including cybergrooming, showed that this program was only effective for adolescents without prior victimization experiences but not for those who were victimized before (Calvete et al., 2023). Therefore, such programs should address experiences and issues that are common among cybergrooming victims. Given the role of depressive symptoms in cybergrooming experiences, as evident in the present study, programs should include basic mental health support. This may not only decrease the risk of victimization in the first place but also help to empower adolescents to cope with negative mental health outcomes following victimization. This suggestion aligns with the findings of the study mentioned above, which showed that an anti-stress intervention comprising coping strategies for stress (e.g., relaxation, cognitive restructuring) was effective in previously victimized adolescents when used as a control condition (Calvete et al., 2023).

Further, building on findings on emotional involvement, prevention programs targeting cybergrooming should include modules that raise awareness about emotional manipulation and how to recognize it. For example, realistic examples of different grooming strategies and their functions can illustrate how perpetrators operate. Additionally, prevention efforts should emphasize that emotional closeness does not preclude a harmful situation. Also, parents and educators should be educated about cybergrooming as a manipulative process that may cause the victim to develop an emotional attachment. This may help them to understand better the victim, their situation, and their actions (e.g., difficulties in ending the “relationship” with the perpetrator), which may ultimately promote a more sensitive and supportive manner of interacting with victims – especially given that some people whom victims of sexual violence confide in react negatively, for example, by blaming them (e.g., Dworkin et al., 2019).

Third, as social support did not have a significant effect on transition odds, general social support might not be enough in preventing young people from being victimized by cybergrooming perpetrators. Rather, aspects of social support that may be relevant to cybergrooming could be decisive, such as ICT-related support. For example, a study showed that instructive parental mediation of Internet use (e.g., guidance on how to use the Internet) was negatively associated with cybergrooming victimization (Wachs et al., 2020). In the context of ICT-related support, pointing out and helping to implement technical options for dealing with the cybergrooming situation could also be relevant, for instance, how to block and report the perpetrator to the platform.

### Limitations

First, the study design did not allow for a differentiation between chronicity of victimization and revictimization, i.e., it was not possible to determine whether individuals were victimized continuously over several months or intermittently. Therefore, no statement can be made regarding whether certain socio-demographic or psychosocial variables are particularly involved in chronicity or others in revictimization. Further, no statements could be made regarding the duration of victimization. For example, one person may have been victimized for a short period shortly before and after T1 and therefore be considered a stable victim, while another person may have been victimized for several months before T1 but not after T1 and therefore not be considered a stable victim. Such individual nuances in victimization experiences can only be assessed using more sophisticated survey methods such as ecological momentary assessment. Finally, experiencing only one or two strategies and thus being classified in the low-victimization class does not necessarily guarantee a “more harmless” form of victimization. For example, for some victims, the transition from the high- to low-victimization class may represent an improvement in victimization severity. On the other hand, for some victims, a sexually exploitative relationship could have been established in which, for instance, sexualization predominates, and there is no longer any interest or deception, resulting in fewer strategies experienced at T2 compared to T1. However, further analyses showed that the number of experienced strategies was associated with the frequency of respective strategies, indicating that a transition to low-victimization was likely to accompany less frequent experiences.

Second, there are some statistical limitations. An LPA did not yield meaningfully interpretable profiles. This was likely because many victims experienced only a few strategies rarely, and the pre-dichotomized mean values of these strategies were therefore heavily right-skewed. For a more robust LPA, a sample with more victims who have experienced the strategies more frequently may be required, but this is only desirable from a statistical perspective. As a result, LCA with dichotomized indicators was performed. While it is still informative, whether someone experienced a certain strategy, and how experienced strategies cluster, dichotomization goes hand in hand with a loss of information and power (MacCallum et al., 2002). Furthermore, the case counts for several transitions in the LTA were low, which impedes robust estimation of the effects of the selected variables on those transitions. In particular, the confidence intervals for some estimated odds ratios were very wide, causing some remarkably high or low estimates to fail to reach statistical significance. Finally, the assignment of victims experiencing three strategies to classes based on their most likely class membership was not consistent over time. More specifically, some patterns were assigned to the low-victimization class at T1 but to the high-victimization class at T2, resulting in a small overlap between the two classes and limiting their distinctiveness and the interpretation of transitions between them. However, by employing the BCH method, the lower classification certainty of cases with these patterns was taken into account in the analyses, which at least partially counteracted the influence of inconsistent classification on the results.

Third, grouping homosexual and bisexual individuals and individuals with other sexual orientations, as well as excluding non-binary individuals from analyses of gender effects, does not adequately reflect sexual and gender diversity. Considering current developments (e.g., threats to LGBTQ+ rights), it is essential that sexual and gender minorities continue to be (and more strongly) considered and acknowledged in research to identify and highlight specific vulnerabilities. However, since the number of sexual and gender minorities was rather small in the present study, they could not be included in the analyses as separate groups from a statistical perspective. Future research should employ a targeted sampling strategy to overcome these issues.

## Conclusion

Young people’s use of and engagement with ICTs is inherently associated with the possibility of encountering various online risks, including cybergrooming victimization. The current body of research on cybergrooming lacks information on potential victim typologies and determinants of victimization stability. The present study, therefore, adopted a person-centered approach to investigate cybergrooming subgroups among adolescents in a longitudinal context. Latent class analysis with experienced grooming strategies as indicators revealed three classes at both time points, namely one class representing non-victims and two victimized classes (low- and high-victimization), which differed primarily quantitatively but not qualitatively. That is, the pattern of experienced strategies was similar, but the probabilities of experiencing them were not. These classes exhibited significant differences regarding gender, age, sexual orientation, depressive symptoms, and emotional involvement, but not social support. Latent transition analysis demonstrated that high stability was particularly evident in the no- and high-victimization classes. Further, transitions between no- and high-victimization classes were influenced by socio-demographic and psychosocial factors, except social support and sexual orientation. This emphasizes that there is a narrow group of adolescents who are vulnerable to high victimization and, moreover, the stability of these victimization experiences. Finally, the findings regarding the role of selected variables indicate that prevention and intervention efforts should also target older adolescents and LGBTQ+ youth and address mental health concerns and emotional manipulation in cybergrooming.

## Supplementary Information

Below is the link to the electronic supplementary material.

Supplementary Material 1

## Data Availability

All materials (data and scripts) are provided in an online repository: https://osf.io/ej53n/files/osfstorage.
