# Supplementary material for: Stability of Cybergrooming Victimization Among Adolescents: A One-Year Latent Transition Analysis
Source: J Youth Adolesc. 2026 Mar 19;55(5):1111–28. doi: 10.1007/s10964-026-02333-w (PMC13156136; doi:10.1007/s10964-026-02333-w)
Supplement: Supplementary file 1 — Supplementary Material 1 [file 10964_2026_2333_MOESM1_ESM.docx]

**Supplementary Material**

Article title: Stability of Cybergrooming Victimization Among Adolescents: A One-Year Latent Transition Analysis

Authors: Catherine Schittenhelm, Manuel Gámez-Guadix, Sebastian Wachs

Corresponding author’s contact information: catherine.schittenhelm@uni-muenster.de

Journal: Journal of Youth and Adolescence

**Table S1**

*Descriptive Statistics of Single Items*

|  |  | **T1** | | |  | **T2** | | |
| --- | --- | --- | --- | --- | --- | --- | --- | --- |
| **Item** | **Description** | ***M* (*SD*)** | **Skew** | **Kurtosis** |  | ***M* (*SD*)** | **Skew** | **Kurtosis** |
| Deceived me into believing they were closer to my age. | Deception 1 | 0.14 (0.46) | 3.91 | 16.68 |  | 0.16 (0.48) | 3.55 | 13.46 |
| Told me they were younger than they actually were. | Deception 2 | 0.16 (0.49) | 3.70 | 14.87 |  | 0.17 (0.48) | 3.30 | 11.98 |
| Deceived me into believing we had things in common or liked the same things. | Deception 3 | 0.13 (0.44) | 3.83 | 15.76 |  | 0.14 (0.45) | 3.48 | 12.77 |
| Pretended to be other people to convince me of things or to scare me. | Deception 4 | 0.07 (0.34) | 5.24 | 30.07 |  | 0.07 (0.33) | 5.20 | 30.99 |
| Offered me free photo sessions. | Gift giving 1 | *excluded* |  |  |  |  |  |  |
| Offered me a job based on my appearance (as a model, public relations, etc.). | Gift giving 2 | *excluded* |  |  |  |  |  |  |
| Offered me money or other things in exchange for sex. | Gift giving 3 | 0.05 (0.32) | 7.67 | 62.11 |  | 0.07 (0.35) | 5.95 | 38.92 |
| Offered me money or other things in exchange for photos or videos of myself. | Gift giving 4 | 0.06 (0.35) | 6.56 | 45.55 |  | 0.07 (0.35) | 6.00 | 39.04 |
| Showed interest in my school schedules, family, etc. | Interest 1 | 0.14 (0.51) | 4.08 | 17.12 |  | 0.11 (0.41) | 4.10 | 18.38 |
| Asked me about my family and friends. | Interest 2 | 0.22 (0.61) | 3.05 | 9.08 |  | 0.18 (0.53) | 3.41 | 12.30 |
| Showed interest in my family problems. | Interest 3 | 0.17 (0.56) | 3.71 | 13.60 |  | 0.15 (0.50) | 3.76 | 14.94 |
| Showed interest in how I was doing, if I was happy. | Interest 4 | 0.28 (0.71) | 2.75 | 6.89 |  | 0.27 (0.68) | 2.78 | 7.21 |
| At first, they didn't talk to me about sex, but later they did. | Sexualization 1 | 0.11 (0.44) | 4.58 | 22.07 |  | 0.13 (0.47) | 4.22 | 18.92 |
| Made jokes or sexual comments to me. | Sexualization 2 | 0.14 (0.51) | 4.06 | 17.07 |  | 0.18 (0.54) | 3.45 | 12.29 |
| Asked me about my sexual experiences. | Sexualization 3 | 0.16 (0.55) | 3.84 | 14.52 |  | 0.20 (0.61) | 3.30 | 10.56 |
| Showed interest in sex from the beginning. | Sexualization 4 | 0.09 (0.39) | 5.19 | 29.42 |  | 0.11 (0.45) | 4.62 | 22.60 |
| Sent me threatening or insulting messages. | Aggression 1 | 0.07 (0.32) | 5.51 | 34.00 |  | 0.08 (0.38) | 5.27 | 29.87 |
| Threatened to spread or post intimate or sexual photos or videos of me on the Internet. | Aggression 2 | *excluded* |  |  |  |  |  |  |
| Wrote or spread jokes, rumors, gossip, or comments that made me look ridiculous. | Aggression 3 | 0.07 (0.36) | 6.15 | 40.65 |  | 0.06 (0.34) | 6.41 | 45.56 |
| Made me look bad in front of my family, friends, or acquaintances. | Aggression 4 | 0.05 (0.30) | 7.51 | 62.41 |  | 0.06 (0.30) | 6.22 | 43.11 |
| Feeling lonely. | Depression 1 | 2.08 (1.17) | 1.03 | 0.23 |  | 2.47 (1.27) | 0.55 | -0.80 |
| Feeling sad. | Depression 2 | 2.55 (1.21) | 0.58 | -0.59 |  | 2.77 (1.27) | 0.34 | -0.94 |
| Losing interest in things. | Depression 3 | 2.40 (1.27) | 0.63 | -0.64 |  | 2.80 (1.31) | 0.27 | -1.08 |
| Feeling hopeless about the future. | Depression 4 | *excluded* |  |  |  |  |  |  |
| Feeling worthless. | Depression 5 | 2.04 (1.29) | 1.09 | -0.01 |  | 2.44 (1.40) | 0.56 | -1.03 |
| Thinking about ending my life. | Depression 6 | 1.36 (0.88) | 2.74 | 6.93 |  | 1.45 (0.96) | 2.33 | 4.74 |
| I have some friends and family members who often encourage me. | Social support 1 | 3.24 (0.98) | -1.45 | 1.89 |  | - | - | - |
| I have some friends and family members who really care about me. | Social support 2 | 3.48 (0.89) | -2.07 | 4.36 |  | - | - | - |
| I always have someone to help me when I need it. | Social support 3 | 3.25 (1.03) | -1.48 | 1.69 |  | - | - | - |
| I have some friends and family members who appreciate my qualities. | Social support 4 | 3.36 (0.92) | -1.72 | 3.06 |  | - | - | - |
| I was so busy with our communication that I had little time for other things. | Emotional involvement 1 | 0.29 (0.64) | 2.44 | 5.74 |  | 0.24 (0.59) | 2.68 | 7.10 |
| He/she wanted to have a romantic relationship with me. | Emotional involvement 2 | *excluded* |  |  |  |  |  |  |
| While we were together, I thought that being part of the relationship was the best and most important thing that had happened to me. | Emotional involvement 3 | 0.25 (0.69) | 2.99 | 8.25 |  | 0.14 (0.43) | 3.44 | 13.26 |
| Gradually, our relationship became more and more important to me. | Emotional involvement 4 | 0.27 (0.64) | 2.65 | 6.93 |  | 0.23 (0.54) | 2.43 | 5.69 |

**Table S2**

*Model Fit of All Tested Measurement Models*

| Measurement Model (*n*) | | χ^2^ | *df* | rCFI | rRMSEA | SRMR | ω | Exclusion due to |
| --- | --- | --- | --- | --- | --- | --- | --- | --- |
| **Time point 1** | | | | | | | | |
| 1a_1_ | Cybergrooming (20) | 305.35 | 160 | .932 | .069 | .083 | [.63; .92] |  |
| 1b_1_ | Cybergrooming (17) | 169.09 | 109 | .974 | .050 | .051 | [.78; .92] | low loadings (3 items) |
| 2a_1_ | Depressive symptoms (6) | 56.12 | 9 | .962 | .106 | .031 | .87 |  |
| 2b_1_ | Depressive symptoms (5) | 15.81 | 5 | .988 | .069 | .020 | .86 | misfit  (1 item) |
| 3a_1_ | Social support (4) | 2.11 | 2 | 1.000 | .009 | .006 | .89 |  |
| 4a_1_ | Emotional involvement (4) | 0.97 | 2 | 1.000 | .000 | .017 | .77 |  |
| 4b_1_ | Emotional involvement (3) | 0.17 | 3 | 1.000 | .000 | .015 | .78 | misfit at T2 (1 item) |
| **Time point 2** | | | | | | | | |
| 1a_2_ | Cybergrooming (20) | 352.00 | 160 | .913 | .073 | .073 | [.68; .91] |  |
| 1b_2_ | Cybergrooming (17) | 172.66 | 109 | .966 | .053 | .055 | [.79; .91] | low loadings (3 items) |
| 2a_2_ | Depressive symptoms (6) | 53.00 | 9 | .972 | .097 | .027 | .89 |  |
| 2b_2_ | Depressive symptoms (5) | 8.97 | 5 | .997 | .039 | .012 | .88 | misfit  (1 item) |
| 3a_2_ | Emotional involvement (4) | 7.77 | 2 | .951 | .165 | .046 | .67 |  |
| 3b_2_ | Emotional involvement (3) | 0.67 | 3 | 1.00 | .000 | .024 | .73 | misfit  (1 item) |

*Note*. *n* = number of indicators, rCFI = robust CFI, rRMSEA = robust RMSEA, ω = McDonald’s Omega. Models for emotional involvement were based on 207 T1-victims and 217 T2-victims. In the second model for emotional involvement (4b_1_, 3b_2_), two loadings were restricted to equality, as otherwise the models would have had zero degrees of freedom, making it impossible to calculate fit indices.

**Table S3**

*Longitudinal Invariance Models Per MOGQ Subscale*

| **Model** | **χ^2^ (df)** | **rCFI** | **rRMSEA** | **SRMR** | **Δ**  **rCFI** | **Δ**  **rRMSEA** | **Δ**  **SRMR** |
| --- | --- | --- | --- | --- | --- | --- | --- |
| **Deception** |  |  |  |  |  |  |  |
| Configural | 13.23 (15) | 1.000 | .000 | .032 |  |  |  |
| Metric | 11.98 (18) | 1.000 | .000 | .032 | 0 | 0 | 0 |
| Scalar | 14.12 (22) | 1.000 | .000 | .033 | 0 | 0 | .001 |
|  |  |  |  |  |  |  |  |
| **Gift giving** |  |  |  |  |  |  |  |
| Configural | 0.000 (1) | 1.000 | .000 | .000 |  |  |  |
| Metric | 0.295 (2) | 1.000 | .000 | .012 | 0 | 0 | .012 |
| Scalar | 0.765 (4) | 1.000 | .000 | .012 | 0 | 0 | 0 |
|  |  |  |  |  |  |  |  |
| **Interest** |  |  |  |  |  |  |  |
| Configural | 25.35 (15) | 0.990 | .056 | .033 |  |  |  |
| Metric | 26.92 (18) | 0.990 | .050 | 0.40 | 0 | -.006 | .007 |
| Scalar | 32.20 (22) | 0.990 | .045 | .041 | 0 | -.005 | .001 |
|  |  |  |  |  |  |  |  |
| **Sexualization** |  |  |  |  |  |  |  |
| Configural | 11.74 (15) | 1.000 | .000 | .022 |  |  |  |
| Metric | 11.91 (18) | 1.000 | .000 | .026 | 0 | 0 | .004 |
| Scalar | 15.06 (22) | 1.000 | .000 | .029 | 0 | 0 | .003 |
|  |  |  |  |  |  |  |  |
| **Aggression** |  |  |  |  |  |  |  |
| Configural | 5.01 (5) | 1.000 | .000 | .029 |  |  |  |
| Metric | 6.19 (7) | 1.000 | .000 | .042 | 0 | 0 | .013 |
| Scalar | 8.63 (10) | 1.000 | .000 | .042 | 0 | 0 | 0 |

*Note*. rCFI = robust CFI, rRMSEA = robust RMSEA. Since the scale for gift giving comprised only two items, the latent factors were correlated with age, allowing the model to have sufficient degrees of freedom to calculate fit indices.

**Table S4**

*Manifest Correlations*

| **Variable** | **1** | **2** | **3** | **4** | **5** | **6** | **7** | **8** | **9** | **10** | **11** | **12** | **13** | **14** |
| --- | --- | --- | --- | --- | --- | --- | --- | --- | --- | --- | --- | --- | --- | --- |
| 1. Deception T1 | - |  |  |  |  |  |  |  |  |  |  |  |  |  |
| 2. Gift T1 | .41** | - |  |  |  |  |  |  |  |  |  |  |  |  |
| 3. Interest T1 | .53** | .38** | - |  |  |  |  |  |  |  |  |  |  |  |
| 4. Sexualization T1 | .53** | .61** | .60** | - |  |  |  |  |  |  |  |  |  |  |
| 5. Aggression T1 | .49** | .38** | .42** | .50** | - |  |  |  |  |  |  |  |  |  |
| 6. Depression T1 | .27** | .14** | .23** | .25** | .24** | - |  |  |  |  |  |  |  |  |
| 7. Social support T1 | -.06 | -.03 | -.06 | -.05 | -.05 | -.27** | - |  |  |  |  |  |  |  |
| 8. Deception T2 | .41** | .07 | .34** | .25** | .28** | .17** | -.03 | - |  |  |  |  |  |  |
| 9. Gift T2 | .40** | .42** | .41** | .48** | .26** | .15** | -.01 | .37** | - |  |  |  |  |  |
| 10. Interest T2 | .33** | .20** | .44** | .40** | .27** | .15** | -.03 | .51** | .33** | - |  |  |  |  |
| 11. Sexualization T2 | .33** | .28** | .39** | .49** | .24** | .15** | -.02 | .53** | .54** | .56** | - |  |  |  |
| 12. Aggression T2 | .25** | .05 | .23** | .23** | .20** | .07 | -.00 | .53** | .31** | .43** | .50** | - |  |  |
| 13. Depression T2 | .19** | .10** | .17** | .17** | .13** | .48** | -.08* | .25** | .13** | .23** | .19** | .13** | - |  |
| 14. Age T1 | .15** | .20** | .20** | .26** | .10** | .17** | -.10* | .08* | .18** | .16** | .25** | .03 | .09* | - |
| 15. Emotional involvement T1 | *.21*** | *.22*** | *.51*** | *.45*** | *.31*** | *.26*** | *-.12* | *.11* | *.28*** | *.32*** | *.28*** | *.19*** | *.20*** | *.12* |
| 16. Emotional involvement T2 | *.19*** | *.03* | *.19*** | *.20*** | *.07* | *.01* | *.04* | *.23*** | *.18*** | *.58*** | *.37*** | *.32*** | *.11* | *.17** |

*Note.* Italic correlations involving emotional involvement are based on 207 T1-victims and 217 T2-victims, respectively. * indicates *p* < .05. ** indicates *p* < .01.

**Instability in Classification**

Results showed a slight inconsistency in classification among those experiencing three strategies. Therefore, patterns of experienced strategies were examined in more detail for participants who experienced three strategies. At T1, 10 such members of the low-victimization class did not experience sexualization. The same applies to 8 members of the low-victimization class at T2, which is why these participants were not assigned to the high-victimization class, as this class was characterized by experiencing sexualization at T1 and T2. Rather, the “problematic” patterns – representing the core of the inconsistency in classification – were those which were assigned to the low-victimization class at T1 but to the high-victimization class at T2. The classification certainty for participants with such problematic patterns was lower, on average, than for the other class members. More specifically, on average, they had lower joint posterior probabilities for their most likely classes (e.g., the probability for being in the low-victimization class at T1 and in the high-victimization class at T2). For example, the mean joint posterior probability for their most likely classes of “problematic” cases in the high-victimization class at T2 was 0.66, while the mean probability for the other class members was 0.88. Using the BCH method, this greater uncertainty in classification was considered in the analyses.

**Designation of Low- and High-Victimization**

First, classes were compared on pre-dichotomized continuous subscale means using the BCH method, i.e., subscale means were incorporated as auxiliary variables in LCA. Table 5 presents the relevant comparison between the low- and high-victimization classes. Results indicated that, on average, all strategies were experienced more frequently in the high-victimization class than in the low-victimization class. It can be argued that the mean values are lower in the low-victimization class, as this class contains more members who have not experienced the respective strategy (i.e., have a subscale mean equal to 0), which depresses the estimated BCH mean. Therefore, low- and high-victimization classes were compared on continuous subscale means, based only on those members who had experienced the respective strategy. However, this had to be done without the BCH method, as only subgroups of the classes were considered, which cannot be implemented within LCA. Instead, means were compared using Welch’s *t*-tests. Here, too, most strategies were experienced more frequently in the high-victimization than in the low-victimization class (see Table 5). These results supported the designation of the classes as low- and high-victimization, as differences between classes were evident beyond the number of experienced strategies.

**Table S5**

*Strategy Means in Low- and High-Victimization Classes*

|  | **Deception** | **Gift giving** | **Interest** | **Sexualization** | **Aggression** |
| --- | --- | --- | --- | --- | --- |
| **Based on all members (BCH)** | | | | | |
| T1 Low-vict. | 0.33 (0.04) | -0.03 (0.02) | 0.58 (0.06) | 0.16 (0.04) | 0.08 (0.03) |
| T1 High-vict. | 0.92 (0.14) | 1.07 (0.19) | 1.36 (0.17) | 1.61 (0.17) | 0.81 (0.14) |
|  |  |  |  |  |  |
| T2 Low-vict. | 0.30 (0.04) | 0.01 (0.02) | 0.33 (0.05) | 0.08 (0.04) | 0.11 (0.03) |
| T2 High-vict. | 0.74 (0.08) | 0.61 (0.11) | 1.11 (0.10) | 1.29 (0.11) | 0.43 (0.09) |
|  |  |  |  |  |  |
| **Based on members who experienced the respective strategy** | | | | | |
| T1 Low-vict. | 0.60 (0.48) | 0.83 (0.58) | 0.83 (0.68) | 0.61 (0.49) | 0.54 (0.41) |
| T1 High-vict. | 0.95 (0.68) | 1.29 (0.94)* | 1.34 (0.83) | 1.42 (0.89) | 1.07 (0.67) |
|  |  |  |  |  |  |
| T2 Low-vict. | 0.53 (0.35) | 0.72 (0.26) | 0.68 (0.52) | 0.61 (0.45) | 0.63 (0.43) |
| T2 High-vict. | 0.78 (0.57) | 1.25 (0.78) | 1.03 (0.68) | 1.11 (0.78) | 0.86 (0.73)* |

*Note. **Every mean difference except these two was significant. Means were calculated for the subscale at the respective time point (i.e., for T1-classes, strategies refer to T1-strategies; analogous for T2-classes).
